# Behavior of Hydrated Lipid Bilayers at Cryogenic Temperatures

**DOI:** 10.3389/fchem.2020.00455

**Published:** 2020-06-18

**Authors:** Jakob Meineke, Martin Weik, Giuseppe Zaccai, Giovanna Fragneto

**Affiliations:** ^1^University of Grenoble Alpes, CEA, CNRS, Institut de Biologie Structurale, Grenoble, France; ^2^Institut Laue-Langevin, Grenoble, France

**Keywords:** flash cooling, lipids, stacked bilayers, neutrons, diffraction, hydration water

## Abstract

Neutron diffraction was used to study the behavior of water present in phospholipid multilamellar stacks from 1,2-dimyristoyl-*sn*-glycero-3-phosphatidylcholine (DMPC) at cryogenic temperatures. Evidence was found for the existence of a highly viscous phase of water that exists between 180 and 220 K based on the observation that water can leave the intermembrane space at these low temperatures. Similar measurements are described in the literature for purple membrane (PM) samples. From a comparison with results from this natural membrane by using the same flash-cooling protocol, it is found that in the case of pure lipid samples, less water is trapped and the water flows out at lower temperatures. This suggests that the water is less hindered in its movements than in the PM case. It is shown that at least the Lβ′-phase of DMPC can be trapped likely by flash cooling; upon heating to about 260 K, it transforms to another phase that was not fully characterized.

## Introduction

The liquid and gel phases of phospholipids and phosphatidylcholines (PCs) have received much attention since the work of (Chapman and Quinn, 1976). Four lamellar phases have been recognized in saturated PCs: a liquid–crystalline phase, Lα, and phases with ordered hydrocarbon chain arrangements, designated ripple phase, Pβ**′**; gel phase, Lβ**′**; and “subgel” or “crystal” phase, Lc.

In the liquid Lα phase, molecules exhibit rotational and lateral diffusion; the transition to the more ordered gel phase is accompanied by a dramatic loss of lateral diffusion and restriction of a variety of bond motions. In this phase, chains are liquid-like and do not present a regular in-plane structure. The Pβ′ phase has been shown to form a periodic surface ripple and exists for all saturated PCs at water contents higher than 20%, in a temperature range just below the main phase transition temperature, Tc (Janiak et al., 1976). In this phase, chains are frozen and tilted with wavelength modulation with a period of ~120–200 Å. In the Lβ′ phase, the chains form a closely packed lattice in which the bilayer planes are flat and tilted with respect to the normal. In the case of bilayers with saturated chains of 16 carbon atoms [1,2-dipalmitoyl-*sn*-glycero-3-phosphatidylcholine (DPPC)], two types of crystal phase have been characterized in which the acyl chains are packed in different subcells, and each phase exhibits a different thermal stability (Ruocco and Shipley, 1982). Differential scanning calorimetry (DSC) heating curves of hydrated DPPC following rapid cooling from 20°C and equilibration at −2°C for increasing periods of time are reported. Immediate reheating shows a small but detectable sub-transition endotherm with transition onset at Tc = −1.6°C. From diffraction and DSC measurements, the authors conclude that that the Lβ′-Lc conversion involves dehydration and hydrocarbon chain ordering (Ruocco and Shipley, 1982).

Neutron and X-ray diffraction from stacks of lipid bilayers, that is, lipid multilayers, are capable of providing key structural information on lipid membranes (Büldt et al., 1979; Zaccai and Gilmore, 1979; Nagle and Tristram-Nagle, 2000; Kučerka et al., 2008). In these experiments, the diffraction of neutrons and X-rays from aligned lipid multilayers deposited on a solid substrate is used to determine the number and nature of the lipid phases present in the sample as well as structural information about the membrane thickness and internal structure (Foglia et al., 2015). In neutron scattering experiments, as well as some NMR or IR spectroscopy experiments, sample deuteration enables highlighting specific membrane components. In neutron scattering experiments, it can also improve the signal-to-noise ratio (Pabst et al., 2010; Luchini et al., 2018).

1,2-Dimyristoyl-*sn*-glycero-3-phosphatidylcholine (DMPC) has been extensively used with neutron techniques as a model lipid for structural and dynamical bilayer studies, the reason being that it is in the fluid phase at room temperature and the deuterated form is easily obtainable in large quantities. The lipid presents a gel-to-gel pre-transition (Lβ′ > Pβ′) at 284.15 K and a gel-to-liquid crystalline main acyl chain crystallization transition (Pβ′ > Lα) at 296.5 K upon temperature increase (Janiak et al., 1979). In heavy water, D_2_O, the transition temperatures are slightly different, being 288.15 K for Lβ′ > Pβ′ and 297.15 K for Pβ′ > Lα (Faure et al., 1997). The *d*-spacing of fully hydrated DMPC samples as determined by X-ray diffraction is reported to be 59.9 Å (Tristram-Nagle et al., 2002). At a relative humidity content <100%, Smith and co-workers (Smith et al., 1990) found that the Lβ′ phase consists in fact of three distinct two-dimensional phases differentiated by the direction of chain tilt with respect to the in-plane lattice. Janiak et al. (1979) discussed also a lamellar structure occurring at low temperature (<0°C) and water content (5–20% w/w) with a similar diffraction pattern and lamellar repeat distance to those found at higher temperatures, suggesting that no major structural alterations occur at the transition from the Lβ′ to this low-temperature phase. DMPC crystallizes from water-containing solution with two water molecules (5% w/w) of hydration, and data have been collected at 10–15°C (Pascher and Pearson, 1979). The crystals are monoclinic (space group P2_1_; unit cell parameters *a* = 8.72, *b* = 8.92, *c* = 55.4 Å and β = 97.40°) with two molecules in the asymmetric unit.

A study of the literature on phospholipid phases at low temperatures indicates a strong dependence on the conditions of hydration and temperature of equilibration of the bilayers so that various phases have been identified like the Pcc phase (so called according to its characteristic convex–concave bilayer curvature observed by freeze-fracture electron microscopy) appearing in DMPC and DPPC samples, when hydration and storage occur at 4°C (Meyer et al., 2000); this phase is not observed when hydration occurs at 50°C and storage at 4°C.

Kiselev et al. (2000) have studied, by small-angle X-ray scattering (SAXS), and wide-angle X-ray scattering (WAXS), ice formation in the presence of cryo-protectants, in model biological membranes formed by phosphocholine lipids including DMPC. Ice formation in fully hydrated DMPC samples occurred at 255.6 K, leading to a decrease in the membrane repeat distance from 59.8 to 53.5 Å. For samples with different degrees of hydration, calorimetric studies (Grabielle-Madelmont and Perron, 1983) allowed the determination of the temperature dependence of the freezing of water and the melting of ice for different water contents in DPPC–water mixtures. The lower the hydration, the lower the temperatures of freezing and melting. More generally, lipid and water dynamics in lipid stacks at subzero temperatures have been studied by temperature-modulated DSC (Svanberg et al., 2009), anelastic spectroscopy (Castellano et al., 2006), Raman scattering (Surovtsev and Dzuba, 2009), and neutron spectroscopy (Swenson et al., 2008). Here, we present a temperature-dependent neutron diffraction study on flash-cooled hydrated DMPC stacks at subzero temperatures and infer about characteristic changes in water and lipid dynamics.

## Materials and Methods

### Sample Preparation for Neutron Diffraction

The sample was prepared from DMPC lipid powder purchased from Avanti Polar Lipids (Alabaster, AL, USA), used without further purification. In 6 ml trifluoroethanol and 2 ml chloroform was dissolved 400 mg of the powder, so that a clear homogenous solution was obtained, which was kept overnight in a freezer at −20°C. The solution was carefully placed into the base of a 4 × 3 cm^2^ flat aluminum sample holder with a pipette, under a glass bowl as protection against dust and other impurities, and left to stand until the solvent was completely evaporated. The evaporation of the solvent and the flat surface of the sample holder favor the formation of multilamellar bilayer stacks (Nagle and Tristram-Nagle, 2000). The sample was then placed in a desiccator and dehydrated under vacuum for 12 h. Finally, it was equilibrated in D_2_O vapor for several days. After equilibration, the sample was tightly sealed with an indium wire placed between the base and the lid, which was then screwed shut. The sample displayed a very high mosaicity, which means that there is not just one single stack of bilayers, all with their bilayer-normal oriented in the same direction, but a number of stacks with an angle of up to 15° between them. This is due to the way in which the sample was prepared.

### Neutron Diffraction, Data Processing, and Analysis

Neutron diffraction experiments were carried out in 2003 on the D16 diffractometer (Leonard et al., 2001; Cristiglio et al., 2015) at the Institut Laue-Langevin (Grenoble, France), with a neutron wavelength λ = 4.53 Å (1% spread, FWHM). The diffracted neutron beam is detected with a square Multi-Wire-Proportional-Chamber ^3^He detector with a resolution of 2 mm in the horizontal and vertical directions. With the detector placed at 1 m from the sample, this corresponds to a resolution of 0.115° (0.0028 Å^−1^ in *Q*, at a wavelength of 4.53 Å) and an angle of 14.7° for the whole detector. Measurements were carried out with a detector–sample distance of 1 m. To ensure that all measurements on one sample were made in the same orientation, the samples were aligned using the rocking-curve method, every time the sample was remounted, for example, after each flash cooling. First, the sample is placed approximately orthogonal to the neutron beam. Then a simple ω-scan, where ω is the sample angle from ω = 70° to 110°, is carried out by observing the intensity of the first lamellar peak, at a detector angle of γ = 5°, corresponding to the lamellar spacing *d* of the membrane stack. This curve is ideally reminiscent of a Gaussian broken by two characteristic minima due to high absorption where the sample is oriented parallel to the incident or diffracted beam. We choose the first minimum to set ω = 90° and measured the position of the lamellar peak at ω = 92°, where the intensity of the rocking curve is maximum. The 2D images were integrated and plotted vs. *Q*, the scattering vector perpendicular to the membrane stacks [*Q* = (4π/λ)sinθ with 2θ being the scattering angle]. Temperature control was carried out in an “Orange” ILL Cryostat on D16 (Cristiglio et al., 2015). Three temperature-dependent data collection protocols were applied sequentially in the following order: (1) slow heating after flash cooling (SHFC protocol), (2) slow cooling (SC protocol), and (3) slow heating (SH protocol).

In the SHFC protocol, the sample was equilibrated for several hours in a cold room at 8°C in order to ensure that the lipids were in the Lβ′ phase. Flash cooling was then achieved by plunging the sample holder into liquid nitrogen in the cold room. Subsequently, the sample holder was transported in liquid nitrogen to D16 and rapidly transferred into the cryostat that had been precooled to 100 K. The temperature was raised from 100 to about 300 K in 5 K steps and was kept constant after each step.

In the SC protocol, the temperature was lowered from 300 to 100 K in 5 K steps and was kept constant after each step. In the SH protocol, the temperature was raised from 100 to 300 K in 5 K steps and was kept constant after each step. The time interval between successive data points was 30 min.

Neutron diffraction data were recorded during the constant-temperature phases. The sample was weighted before and after each neutron diffraction experiment in order to ascertain that the sample was properly sealed and did not lose hydration water in the vacuum of the cryostat during diffraction experiments.

**Figure 1:**
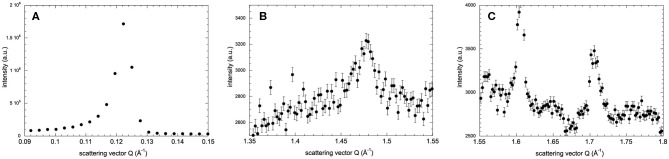
Neutron diffraction intensity of hydrated DMPC lipid stacks at 260 K as a function of the scattering vector *Q* in three different *Q*-ranges. Diffraction peaks originate from **(A)** the lamellar spacing, **(B)** in-plane lipid ordering, and **(C)** crystalline water ice. In **(C)**, the peaks at *Q* = 1.61 Å^−1^ and *Q* = 1.71 Å^−1^ are called *ice peak 1* and *ice peak 2*, respectively. Diffraction intensities in all three *q*-ranges have been recorded after flash cooling upon heating from 100 to 300 K (SHFC protocol). Error bars in **(B,C)** correspond to sqrt(*N*(*x, y*))/*t*, where *N*(*x, y*) are the total counts in pixels *x, y* of the detector and *t* is the acquisition time. In **(A)**, the error bars are too small to be seen.

**Figure 2:**
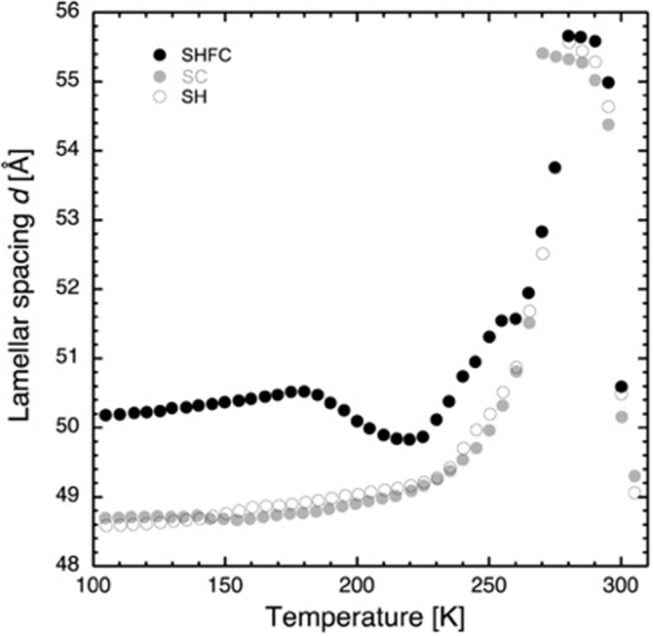
Lamellar spacing of stacks of hydrated DMPC lipids as a function of temperature as determined by neutron diffraction on D16. Closed black circles show the lamellar spacing after flash cooling upon heating from 100 to 300 K (SHFC protocol), closed gray circles represent the ones during subsequent slow cooling from 300 to 100 K (SC protocol), and open circles show the ones during subsequent slow heating from 100 to 300 K (SH protocol). The time interval between successive data points was 30 min. The experimental sequence of heating and cooling steps started with flash cooling, followed by SHFC, then SC, and finally SH.

**Figure 3:**
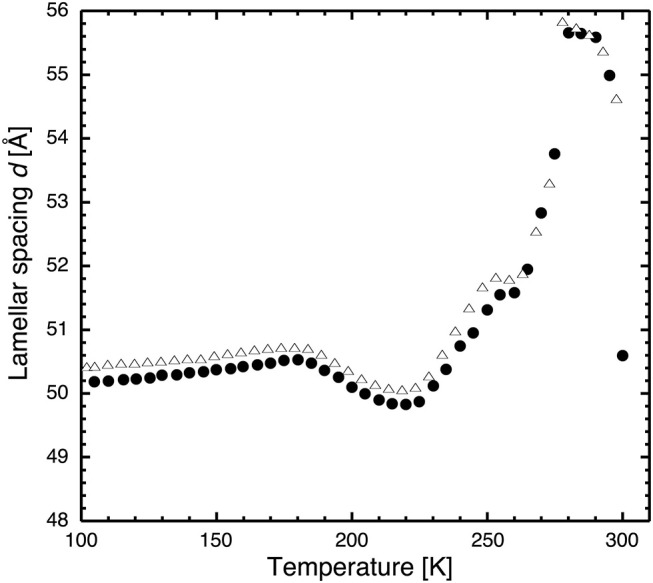
Lamellar spacing of stacks of hydrated DMPC lipids as a function of temperature as determined by neutron diffraction on D16 after flash cooling upon heating from 100 to 300 K (SHFC protocol). Closed circles correspond to the data presented in Figure 2, open triangles to those resulting from a repeated experiment carried out with the same sample. The similarity of the two curve profiles informs about the reproducibility of the experiment.

**Figure 4:**
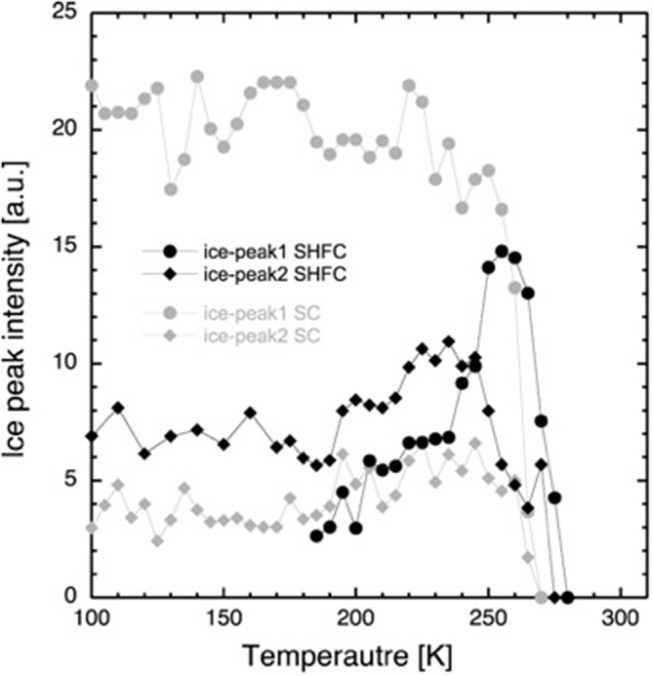
Integrated intensity of the diffraction peaks at *Q* = 1.61 Å^−1^ (*ice peak 1*, circles) and *Q* = 1.71 Å^−1^ (*ice peak 2*, diamonds) after flash cooling upon heating from 100 to 300 K (SHFC protocol, black data points) and during subsequent slow cooling from 300 to 100 K (SC protocol, gray data points). The time interval between successive data points was 30 min.

**Figure 5:**
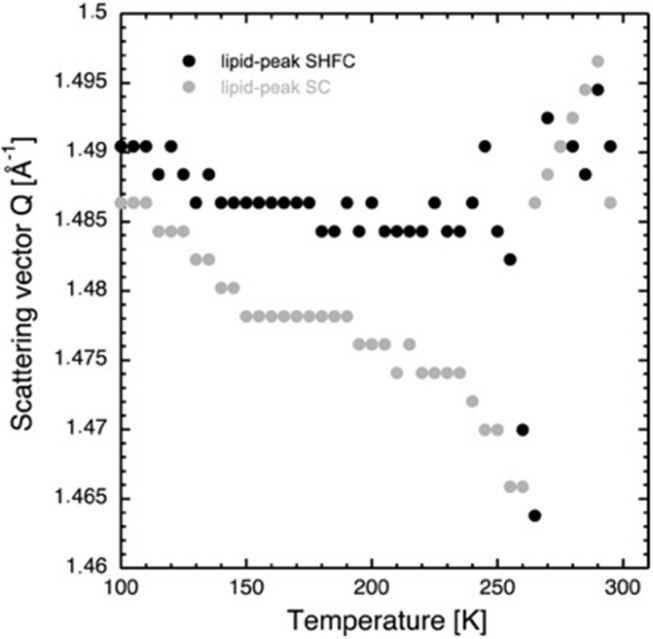
Scattering vector *Q* of the *lipid peak* as a function of temperature during the SHFC protocol (black circles) and the subsequent SC protocol (gray circles).

The lamellar spacing, *d*, was determined from the position of the first-order Bragg peak of the lipid stack (Figure 1A) in an experimental and reversible series consisting of sequential executions of the SHFC, SC, and SH protocols (Figures 2, 3). To this end, the sample had been aligned with an angle of 92° between the normal of the sample and the incoming neutron beam and data recorded for 10 min at each temperature plateau. In a second series of sequential executions of the SHFC and SC protocols, a diffraction peak (called *lipid peak* hereafter; Figure 1B) originating from the intra-planar molecular structure of the phase Lβ′ of the lipids (scattering vector *Q* = 1.49 Å^−1^ at 300 K) and ice formation (Figure 1C) were monitored simultaneously. Transmission geometry was used for these measurements. The sample was aligned with an angle of 32.5° and 35.5° between the normal of the sample and the incoming neutron beam to measure the lipid peak and the ice peaks, respectively, and data were recorded for 7 min at each angle and temperature plateau. Ice formation was monitored (Figure 4) by integrating the intensities of part of the most prominent powder ring, which corresponds to spacing of 3.91 Å [*Q* = 1.61 Å^−1^; (1 0 0) reflection of hexagonal ice; called *ice peak 1* hereafter] and 3.67 Å [*Q* = 1.71 Å^−1^; originating from amorphous ice, the (1 1 1) reflection of cubic ice, or the (0 0 2) reflection of hexagonal ice; called *ice peak 2* hereafter; Figure 1C]. Figure 5 shows the scattering vector *Q* of the *lipid peak* (Figure 1B) as a function of temperature.

## Results

### Lamellar Spacing of the Lipid Stack as a Function of Temperature

After flash cooling, at 100 K, the lamellar spacing *d* is 50.2 Å. In the literature, the bilayer thickness is given to be 44.5 Å in the gel phase (Janiak et al., 1976) and 39 Å in the fluid phase (Perino-Gallice et al., 2002). A lipid contraction of about 1–2% for membranes between 300 K and liquid nitrogen temperature may be deduced from the literature (Weik et al., 2005; Mehra et al., 2020) which, in our case, leads to a bilayer thickness of 43.8 ± 0.2 Å and therefore a water layer with a thickness of 6.4 ± 0.2 Å separating adjacent bilayers in the stack. The reason for using values from the literature for the bilayer size is that it is not possible to perform contrast variation studies on the same sample in these experiments that would allow the mathematical calculation of the bilayer thickness. Furthermore, at least three lamellar peaks would be needed for such a determination (Leonard et al., 2001), and only two were observed in this work.

Upon heating (SHFC protocol) to 180 K, *d* increases to 50.5 Å and then decreases to 49.8 Å at 220 K. Further heating leads again to an increase in d, with a plateau between 255 and 260 K, a maximum of 55.7 Å at 280 K, and a sudden drop at 290 K. During the following slow cooling (SC protocol), the evolution of *d* globally follows the one during the preceding SHFC protocol down to 265 K and then continues to decrease to reach a *d* value of 48.7 Å at 100 K. During subsequent heating (SH protocol) to 300 K, *d* evolves almost identically to the preceding SC protocol with the exception of a hysteric behavior between 270 and 280 K.

### Ice Formation in the Interlamellar Space as a Function of Temperature

After flash cooling, at 100 K, the absence of *ice peak 1* and the presence of *ice peak 2* indicate the absence of hexagonal and the presence of cubic and/or amorphous ice, respectively. Upon heating (SHFC protocol), the integrated intensity of *ice peak 2* remains more or less constant up to 190 K (Figure 4). Between 190 and 240 K, the integrated intensity of *ice peak 2* increases, and *ice peak 1* appears and increases in intensity. Upon further heating from 240 to 300 K, the integrated intensity of *ice peak 2* decreases to zero, and that of *ice peak 1* continues to increase, reaches a maximum at 260 K, and then decreases to zero. Upon subsequent slow cooling (SC protocol), *ice peak 1* appears and increases in intensity at a high rate between 270 and 250 K and at a lower rate between 250 and 220 K and then fluctuates upon further cooling to 100 K. *Ice peak 2* appears and increases in intensity in parallel to *ice peak 1* down to 245 K and then slightly decreases in intensity during further cooling to 100 K. When comparing results from the SHFC and the SC protocols, it is evident that *ice peak 1* intensities are higher than *ice peak 2* intensities during cooling and lower during heating.

### Changes in the Lipid Phase as a Function of Temperature

After flash cooling, the position of the lipid peak (Figure 1B) slightly shifts to lower *Q* values during heating from 100 to 240 K (SHFC protocol, Figure 5). A sudden shift to even lower *Q* values is observed between 240 and 265 K, a temperature interval that includes the plateau observed in the evolution of the lamellar spacing *d* in the SHFC experiment (Figure 2). Upon further heating to 290 K, the lipid peak position shifts to higher *Q* values (Figure 5). During the subsequent slow cooling (SC protocol), the shift in the lipid peak position follows down to 260 K that of the previous SHFC experiment. Upon further cooling from 260 to 100 K, the *Q* value of the lipid peak position increases again and reaches 100 K, a similar value to that after flash cooling.

## Discussion

Based on their temperature-dependent behavior and their position, the three observed Bragg peaks at high angles (for all measurements) were attributed to the Lβ-phase of the lipid (Figure 1B) and to the hexagonal and the cubic forms of the ice in the sample (Figure 1C). The growth of hexagonal ice from cubic ice could be observed starting from around 230 K where *ice peak 1* grows considerably, while *ice peak 2* remains constant.

Based on a value of 44.5 Å for the bilayer thickness (Janiak et al., 1976), we can calculate an approximate value for the thickness of the water layer by subtracting this value from the *d*-spacing. For 100 K after SC, we have about 5.7 Å, which corresponds to roughly two layers of water (Lechner et al., 1998). These two layers are the first hydration layers of the lipids that do not crystallize at subzero temperatures (Castellano et al., 2006). Because of the high affinity of the water molecules to the lipid head groups, the diffusion of water in these layers is considerably slowed down. At 280 K, the water layer thickness is ≈12 Å.

The increase in *d*-spacing when heating from 100 to 180 K could have been caused by an increase in bilayer thickness or an increase in the volume taken up by the water between the bilayers or by both. We observe no change in the position of *ice peak 2* and only a slight change in the lipid peak position. It is thus reasonable to assume that the lipid bilayer is the cause for the increase and that the water density remains constant. It has been reported that the volume per lipid increases with rising temperature in a linear manner within one phase (Nagle and Tristram-Nagle, 2000). Even though the reported measurements were carried out above 273 K, there is no reason to suspect a different behavior at cryo-temperatures.

Now we turn to the decrease in *d*-spacing that we observe in the temperature window from 180 to 220 K on heating a flash-cooled sample (Figure 2), the central part of our study. Several scenarios are possible to explain the decrease.

First, the lipid bilayer undergoes a transition that diminishes its thickness. Secondly, the water density increases, without any change in the state of the lipids. Thirdly, neither lipid bilayer nor the water density changes, and a portion of the water leaves the inter-bilayer space. From the raw diffraction data, it is observed that in the range 180–220 K, the lipid peak (Figure 1B) does not change abruptly, neither in shape nor in intensity and, most importantly, not in position. Since the lipid peak position and the area per lipid molecule and the bilayer thickness are correlated, we conclude that the bilayer does not become thinner and the first scenario can be discarded. The second seems very unlikely, since we observe no change in the position of the ice peaks that would be expected if ice density increased. In addition, such a change would correspond to a transition to another non-equilibrium state on heating. This is not likely, since the molecules' ability to return to equilibrium is increased when increasing thermal energy. The third scenario is supported by the observation of a growth in ice peak intensity (Figure 4). It is widely accepted that crystalline ice does not form within the lipid bilayers (Gleeson et al., 1994); therefore, the observed increase in ice peak intensity must be due to ice forming outside of the membranes. All water not being confined by the membranes must surely be frozen at these temperatures, so the water forming the newly observed crystalline ice is likely to be the interlamellar water that has started to leave the bilayers at 180 K. The water leaving the bilayer space at 180 K is reminiscent of the ultra-viscous water in the no-man's land reported earlier (Mishima and Stanley, 1998). We note that the glass transition of water confined in dioleoylphosphatidylcholine (DOPC) membranes has been reported to take place at a similar temperature, that is, at 170 K (Castellano et al., 2006). A decrease in *d*-spacing on heating a flash-cooled sample to above 180 K has occurred reproducibly in the same sample in three different experiments (see, for example, Figure 3). A comparison with the decrease in *d* observed for a similar experiment with purple membranes (PMs) (Weik et al., 2005) shows that the decrease in *d* is considerably lower here, which suggests that by using the same flash-cooling procedure, that is, the same cooling rate, a significantly lower amount of water can be trapped in the process. This, together with the fact that the water in DMPC leaves the intermembrane space at a temperature (180 K) that is 20 K lower than in PM (200 K), suggests that the interlamellar water is less hindered in its movements by the pure-lipid bilayers than by the PM. Water flowing out of the intermembrane space above 180 K (Figure 2), forming crystalline ice (Figure 4) is reminiscent of cold crystallization (Tanaka et al., 2000) of intermediate water (Tanaka et al., 2015) associated with polymers.

Coinciding with the increase of *d* above 230 K (Figure 2), a dramatic decrease in the intensity of *ice peak 2* is observed (Figure 4). We interpret this decrease as melting of cubic ice that most probably had formed during flash cooling outside of the interlamellar space (Weik et al., 2005). At the same time that the intensity of *ice peak 2* decreases, that of *ice peak 1* increases (Figure 4), indicating the formation of hexagonal ice. Above 230 K, we thus suggest that the melting cubic ice partially recrystallizes into hexagonal ice outside the membrane stack and partially rehydrates the membrane stack, leading to the increase in *d* (Figure 2). In contrast to the decrease in *d* from 180 to 220 K, the plateau from 250 to 265 K is accompanied by a drastic change in the position of the in-plane lipid peak (Figure 5). This suggests that a transition Lβ′-phase appears to be trapped by flash cooling since the position in *Q* of the lipid peak is similar before and after flash cooling (see Figure 5). The trapped Lβ′-phase then probably transforms into another lipid phase at around 260 K. The fact that this plateau occurs only when examining a flash-cooled sample could mean that in addition to the water, a lipid phase was trapped during flash cooling that normally does not exist at these temperatures. In this interpretation, the plateau and the subsequent identical evolution of *d* in SHFC and SH experiments mean the lipid phase trapped by flash cooling returns to equilibrium at around 260 K. The temperatures where trapped water (180 K) and trapped lipids (260 K) gain mobility are different, in line with observations in single supported DMPC bilayers that suggested a decoupling of hydration water and membrane freezing (Toppozini et al., 2012).

Regarding the SH experiment (Figure 2), the increase in *d* during heating from 100 to 225 K is again likely to be due to a change in the lipid organization. Above around 230 K, *d* increases at a higher rate, again suggesting membrane rehydration. The hysteresis in *d* observed when comparing SH and SC experiments is very similar to the one observed by Gleeson et al. (1994), indicating the presence of super-cooled water during SC.

In order to rationalize the draining of water from the intermembrane space upon SC and water influx upon heating, one may consider that the chemical potential of water confined by the bilayers depends strongly on the thickness of the water layer (Gleeson et al., 1994). When the thickness of the water layer *d*_*w*_ is smaller than 10 Å, which is the case at subzero temperatures considering a DMPC bilayer thickness of around 44.5 Å (Janiak et al., 1976) (cf. Figure 2), this dependence is well-approximated by an exponential law so that the difference of the chemical potential is Δμ = *C* ^*^ exp(–*d*_*f*_/Λ), where *C* is a scale factor and Λ the hydration repulsion decay length (Gleeson et al., 1994). The chemical potential of the sample is given by the known chemical potential μ_T_ of ice existing in equilibrium after formation in pockets and defects of the sample when the temperature is about 265–270 K and is then imposed on the interlamellar water. μ_T_ changes with temperature and bilayer thickness changes, which means water flows in or out of the membrane, so that μ inside and outside of the membranes match again.

In the SHFC experiment, the sample is in a metastable state after flash cooling. The water in the flash-cooled system did not form hexagonal ice. From the fact that the intensity of *ice peak 2* in SHFC is almost double than that of *ice peak 2* in the SC experiment, we can infer that a high amount of cubic ice has been formed during flash cooling. Most likely, all ice present at 100 K after flash cooling is cubic ice, present in defects where it is confined enough to have not been able to form hexagonal ice. The (1 1 1) reflection of cubic ice is of a higher order than the (1 0 0) reflection from hexagonal ice. This might explain why the intensity of *ice peak 2* in SHFC is much lower than that of *ice peak 1* in SC, even though both peaks represent about the same amount of ice.

## Conclusions

We studied the behavior of a bilayer stack of synthetic phospholipids (DMPC) in a hydrated environment, at cryo-temperatures, and present evidence for the existence of a highly viscous phase of water above 180 K in flash-cooled samples. In comparison with PMs (Weik et al., 2005), less water is trapped using the same flash-cooling protocol, and the water flows out at lower temperatures, which suggests that the water is less hindered in its movements than in PMs. Our results suggest it is likely that at least the Lβ′-phase of DMPC can be trapped by flash cooling and that it transforms at around 260 K to a different phase, which was not fully characterized.

## Data Availability Statement

All datasets generated for this study are included in the article/supplementary material.

## Author Contributions

JM, GF, and MW designed and performed the experiments. JM and MW did the data analysis. GF, GZ, and MW wrote the manuscript. All authors contributed to the article and approved the submitted version.

## Conflict of Interest

The authors declare that the research was conducted in the absence of any commercial or financial relationships that could be construed as a potential conflict of interest.
